# Author Correction: Multi-omic and single-cell profiling of chromothriptic medulloblastoma reveals genomic and transcriptomic consequences of genome instability

**DOI:** 10.1038/s41467-025-56164-7

**Published:** 2025-01-27

**Authors:** Petr Smirnov, Moritz J. Przybilla, Milena Simovic-Lorenz, R. Gonzalo Parra, Hana Susak, Manasi Ratnaparkhe, John KL. Wong, Verena Körber, Jan-Philipp Mallm, George Philippos, Martin Sill, Thorsten Kolb, Rithu Kumar, Nicola Casiraghi, Konstantin Okonechnikov, David R. Ghasemi, Kendra Korinna Maaß, Kristian W. Pajtler, Anna Jauch, Andrey Korshunov, Thomas Höfer, Marc Zapatka, Stefan M. Pfister, Wolfgang Huber, Oliver Stegle, Aurélie Ernst

**Affiliations:** 1 Group Genome Instability in Tumors, German Cancer Research Center (DKFZ) and German Cancer Consortium (DKTK), Heidelberg, Germany; 2 European Molecular Biology Laboratory, Genome Biology Unit, Heidelberg, Germany; 3 Division of Computational Genomics and Systems Genetics, German Cancer Research Center (DKFZ), Heidelberg, Germany; 4 Wellcome Sanger Institute, Wellcome Trust Genome Campus, Cambridge, UK; 5 Life Sciences Department, Barcelona Supercomputing Center, Barcelona, Spain; 6 Division of Molecular Genetics, German Cancer Research Center (DKFZ), Heidelberg, Germany; 7 Division of Theoretical Systems Biology, German Cancer Research Center (DKFZ), Heidelberg, Germany; 8 Single-cell Open Lab, German Cancer Research Center (DKFZ) and Bioquant, Heidelberg, Germany; 9 Faculty of Biosciences, Heidelberg University, Heidelberg, Germany; 10 Hopp Children’s Cancer Center Heidelberg (KiTZ), Heidelberg, Germany; 11 Division of Pediatric Neuro-oncology, German Cancer Consortium (DKTK) and German Cancer Research Center (DKFZ), Heidelberg, Germany; 12 Department of Pediatric Oncology, Hematology and Immunology, Heidelberg University Hospital, Heidelberg, Germany; 13 Institute of Human Genetics, Heidelberg University, Heidelberg, Germany; 14 Clinical Cooperation Unit Neuropathology, DKFZ, Department of Neuropathology, Heidelberg University Hospital, Heidelberg, Germany

**Keywords:** Cancer genomics, Tumour heterogeneity, CNS cancer

Correction to: *Nature Communications* 10.1038/s41467-024-54547-w, published online 23 November 2024

In this article the funding from ‘German Federal Ministry of Education and Research’ was omitted. The original article has been updated.

