# Supplementary material for: Multi-omic and single-cell profiling of chromothriptic medulloblastoma reveals genomic and transcriptomic consequences of genome instability
Source: Nat Commun. 2024 Nov 23;15:10183. doi: 10.1038/s41467-024-54547-w (PMC11585558; doi:10.1038/s41467-024-54547-w)
Supplement: Supplementary file 1 — Supplementary Information [file 41467_2024_54547_MOESM1_ESM.pdf]

Supplementary Information

Supplementary Figure 1

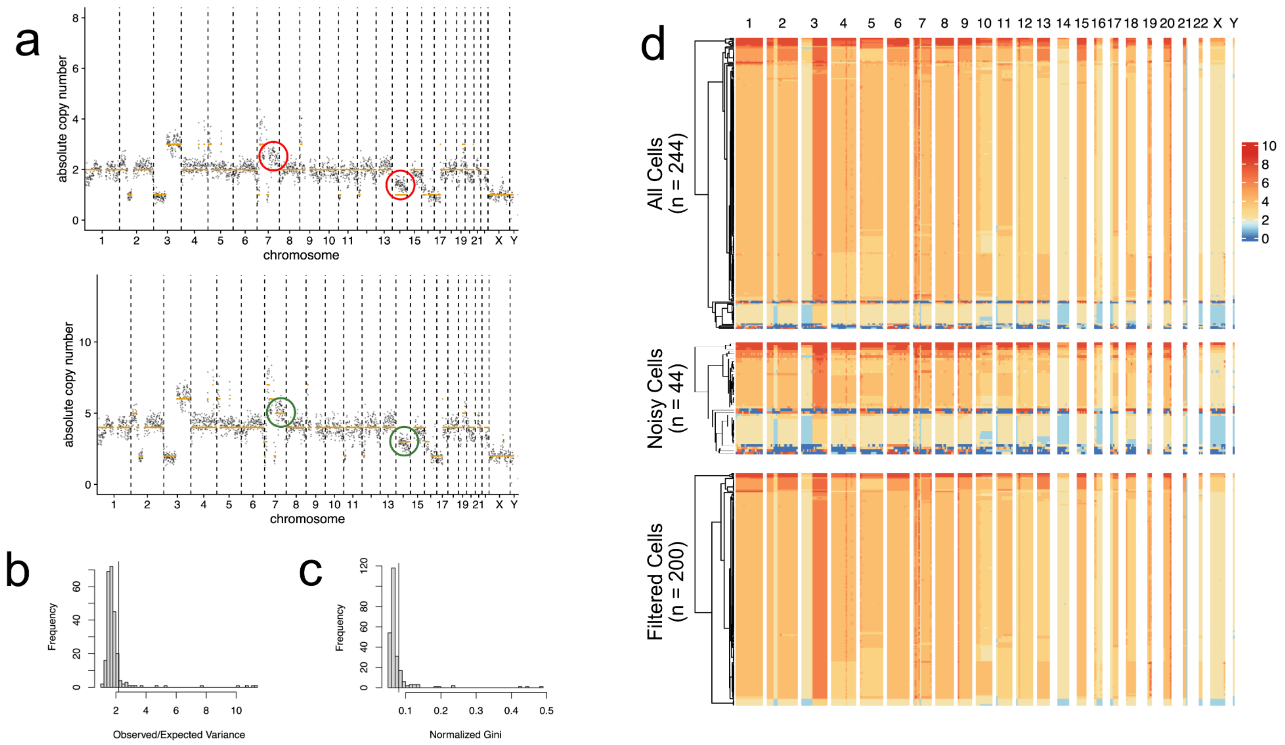

Supplementary Figure 1. Single-cell data quality checks and filtering criteria.
a, top panel, an example of a cell from the LFS MBP Nuclei sample where scAbsolute initially picked a ploidy 2 solution, but large segments of chromosomes had read counts lying clearly in between integral copy number states (circled in red), with the ploidy 4 solution plotted before, showing the better fits to the same regions (circled in green).
b, histogram of the observed/expected variance ratio across bins, with the threshold used for filtering (2 median absolute deviations from the median) shown by the vertical line.
c, same as in b, but displaying the normalised gini coefficient.
d, heatmap for 244 cells from the LFS MBP PDX sample, 44 cells filtered out due to overdispersion or high normalised Gini coefficient, and 200 cells used in the subsequent analysis.
Source data are available with this manuscript.

Supplementary Figure 2

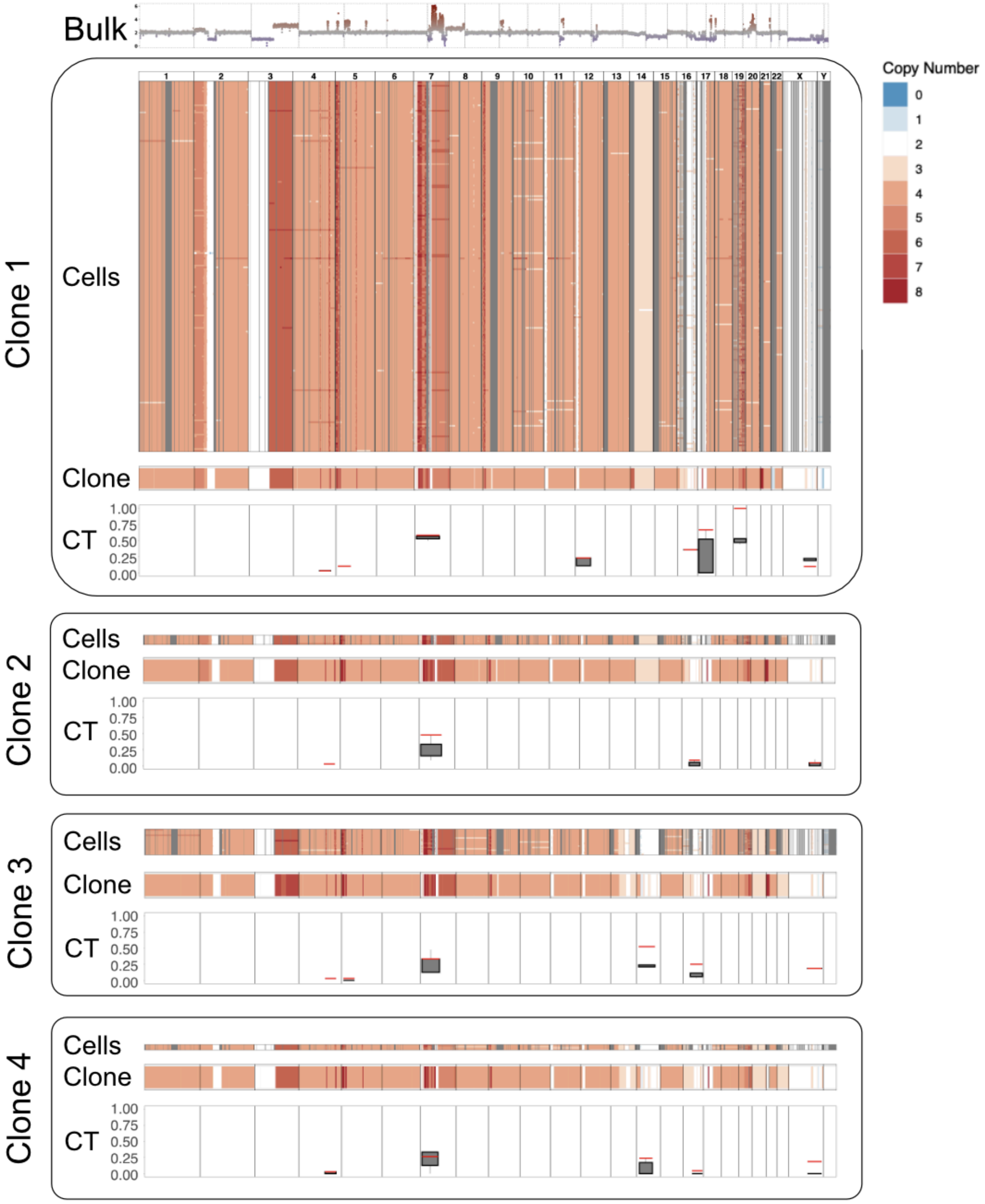

Supplementary Figure 3

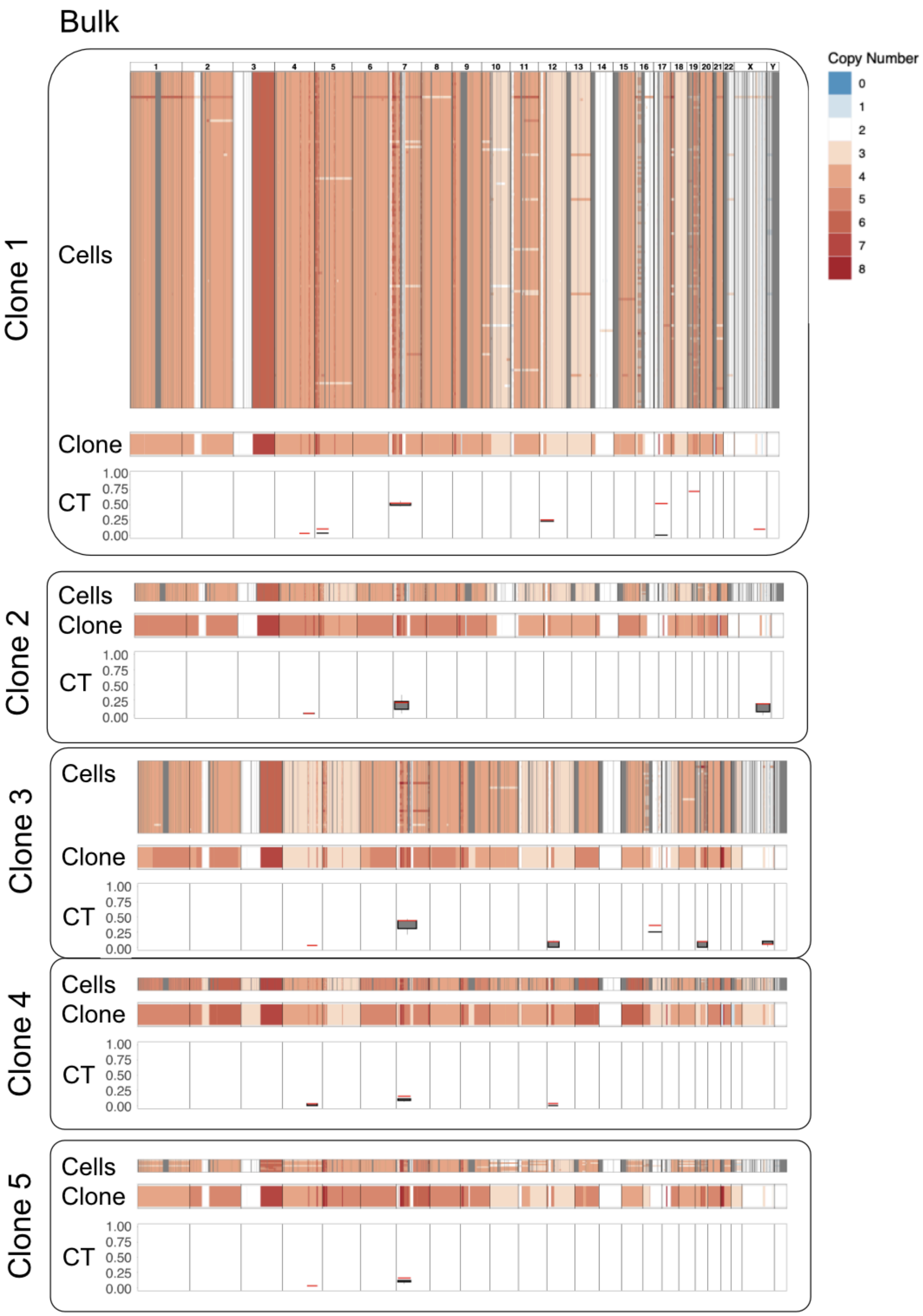

Supplementary Figure 4

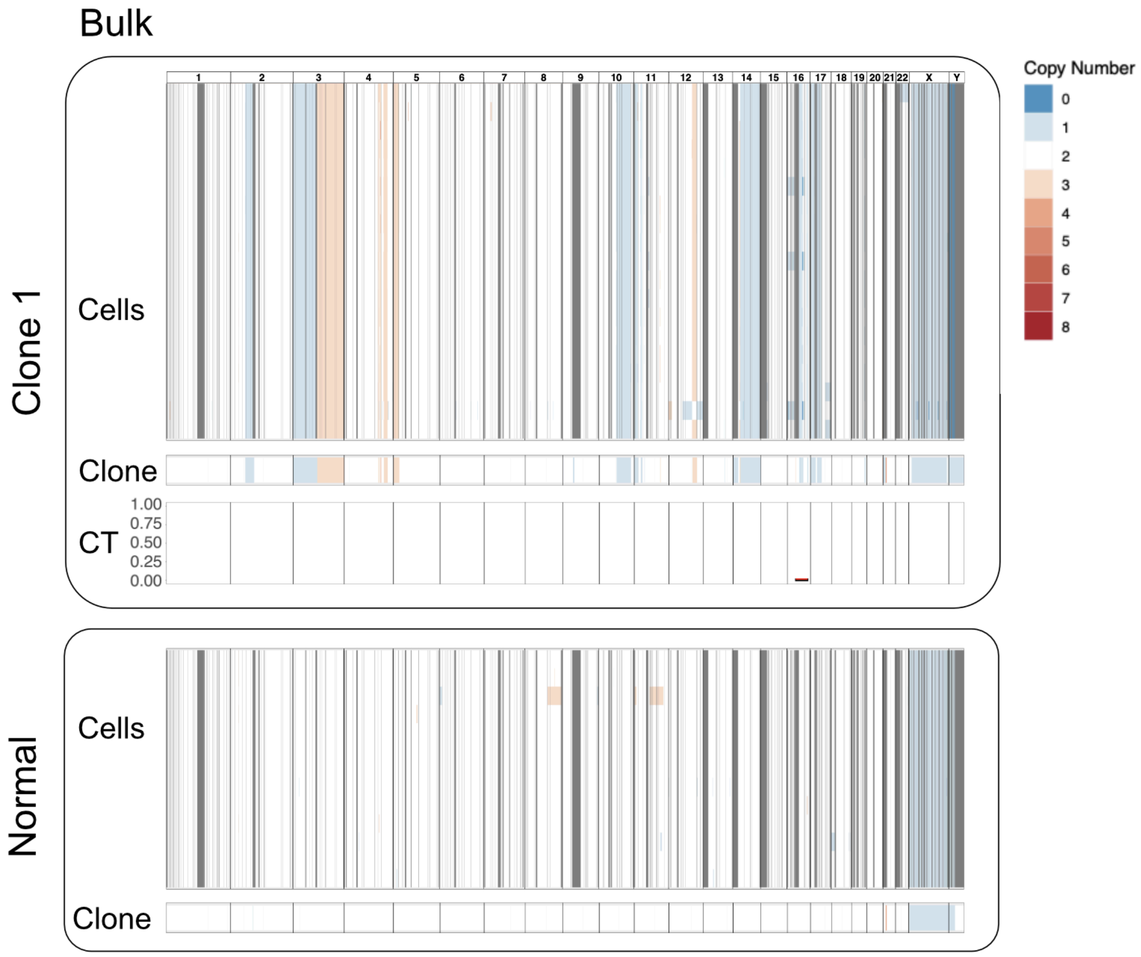

Supplementary Figure 5

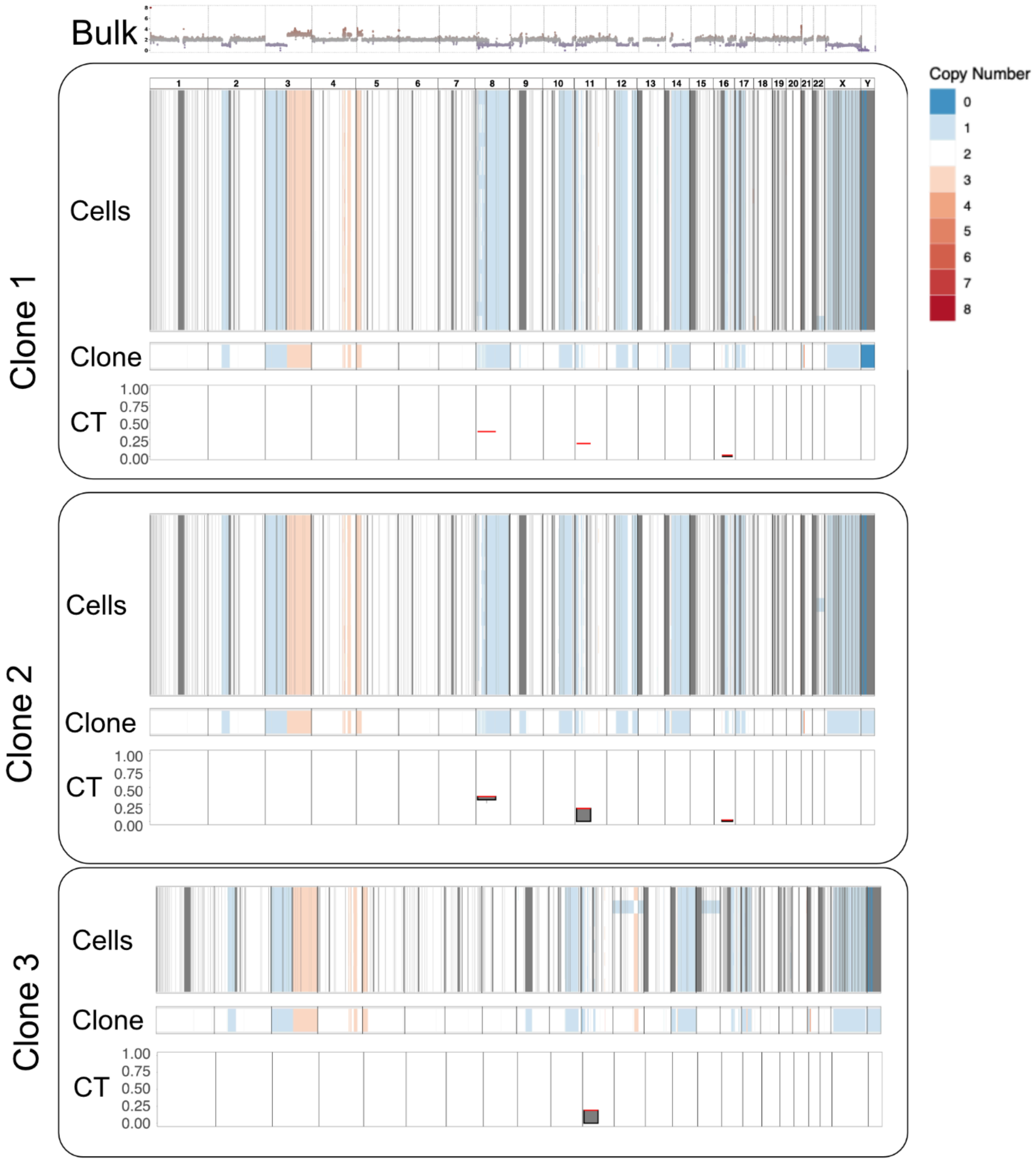

Supplementary Figure 6

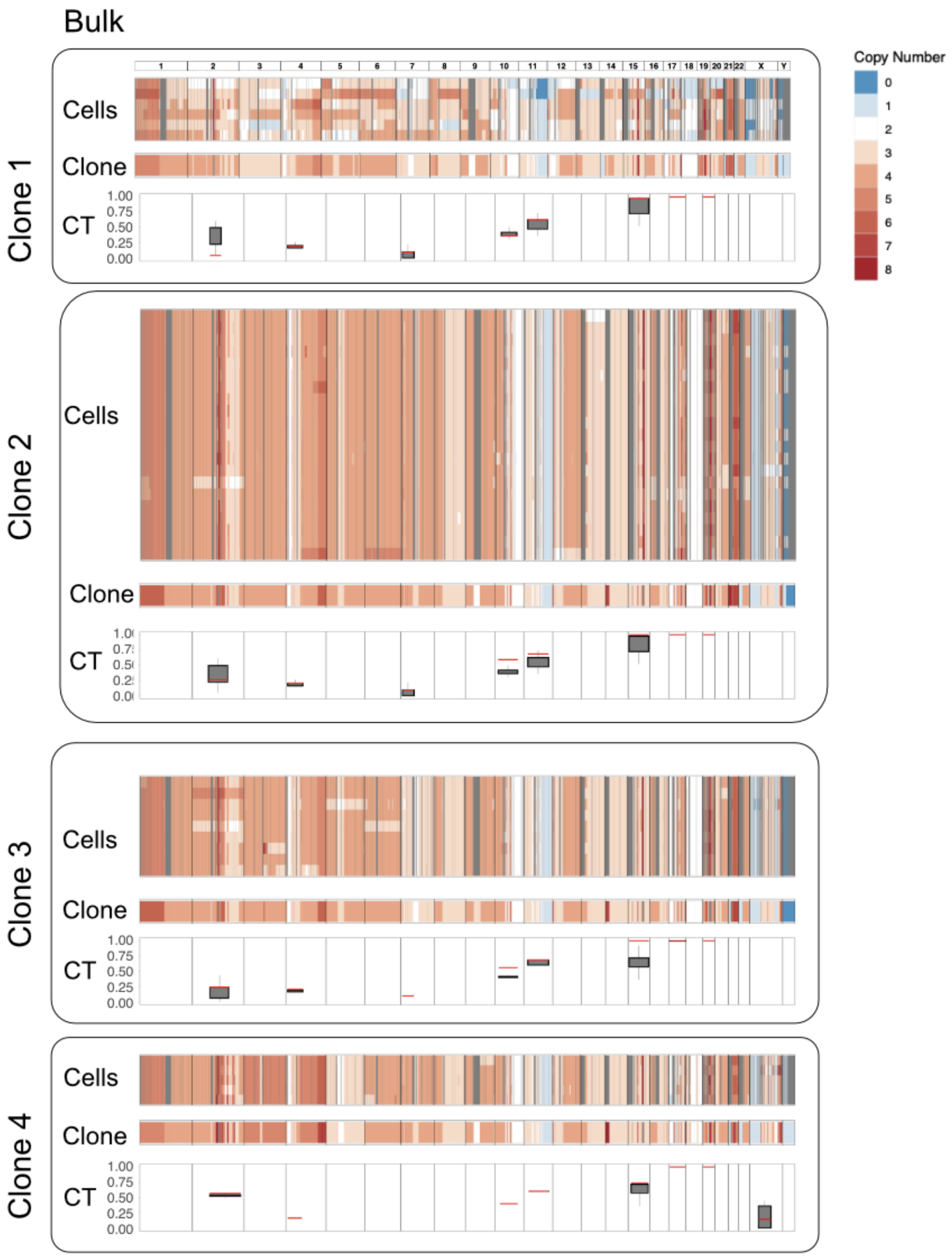

Supplementary Figure 7

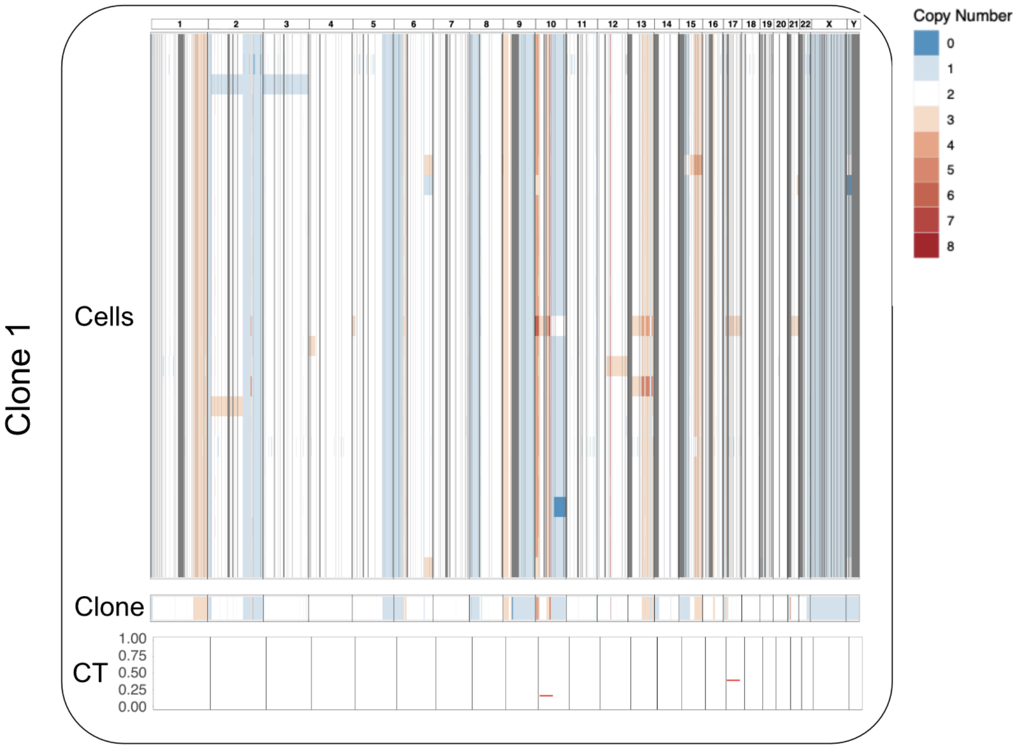

Supplementary Figures 2-7. Copy number heatmaps and chromothripsis scores for all samples profiled by 10X scDNA-seq. From top to bottom: somatic copy number profiles from bulk DNA-seq (Bulk) when available, then for each clone: per-cell copy number profiles as inferred by scAbsolute; pseudobulk copy-number profiles as inferred with HMMCopy for each clone, and chromothripsis scores (fraction of chromosome overlapping chromothriptic regions; Methods) from the observed data (red line) and from 101 bootstrapped clones (Tukey boxplot). **Supplementary Figure 2:** LFS MBP Nuclei; **Supplementary Figure 3:** LFS MBP PDX; **Supplementary Figure 4:** LFS MB1R Nuclei; **Supplementary Figure 5:** LFS MB1R PDX; **Supplementary Figure 6:** MB243 Nuclei; **Supplementary Figure 7:** RCMB18 PDX.

Source data are available with this manuscript.

Supplementary Figure 8

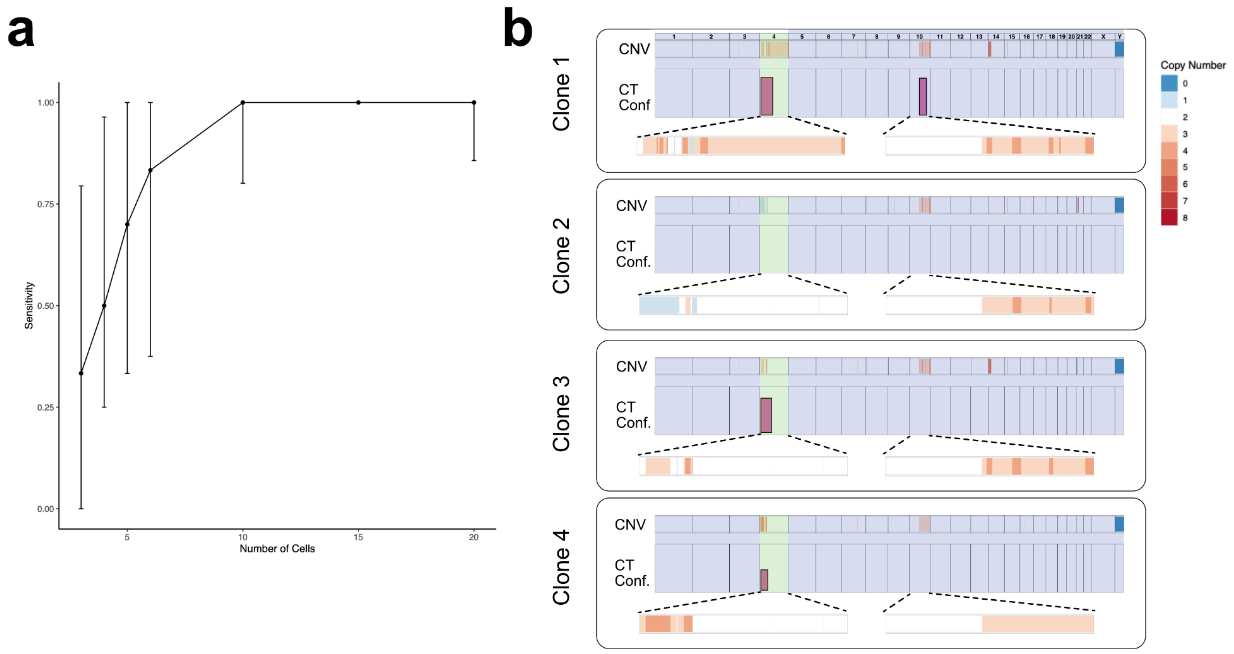

Supplementary Figure 8. a, Sensitivity analysis of detecting CT events assessed using subsampling. Each point is the median sensitivity for detecting CT events at this number of cells per clone across each clone and CT event in our data. Sensitivity is calculated for each individual event as the number of times the event is detected when randomly downsampling the cells assigned to the corresponding clone 101 times, followed by CNV inference and CT scoring of the resulting pseudo bulk read count profiles (Methods). Error Bars show Interquartile Range across events and clones. Only events that were observed in clones with at least the number of cells under consideration for each point were included in the calculation of the median and IQR. **b,** Clonal CNV profiles and CT scores for cells from Clone 2a in Umbreit et al. [PMID: 32299917][1], where a complex genotype was artificially induced using CRISPR-CAS9 targeted to chromosome 4. Our analysis pipeline identified 4 subclones in this population of cells (Supplementary Data 3), and detected CT as expected on chromosome 4 (highlighted in green) in 3/4 major subclones detected in the dataset, and on chromosome 10 in the case of a single clone. Assuming that the CRISPR induced CT occurred only on chromosome 4 as expected, and treating the remaining chromosomes (in blue) as the negative class, the specificity of our CT detection approach would be 91/92 non-CT chromosomes called as non-CT (99%). Source data are available with this manuscript.

Supplementary Figure 9

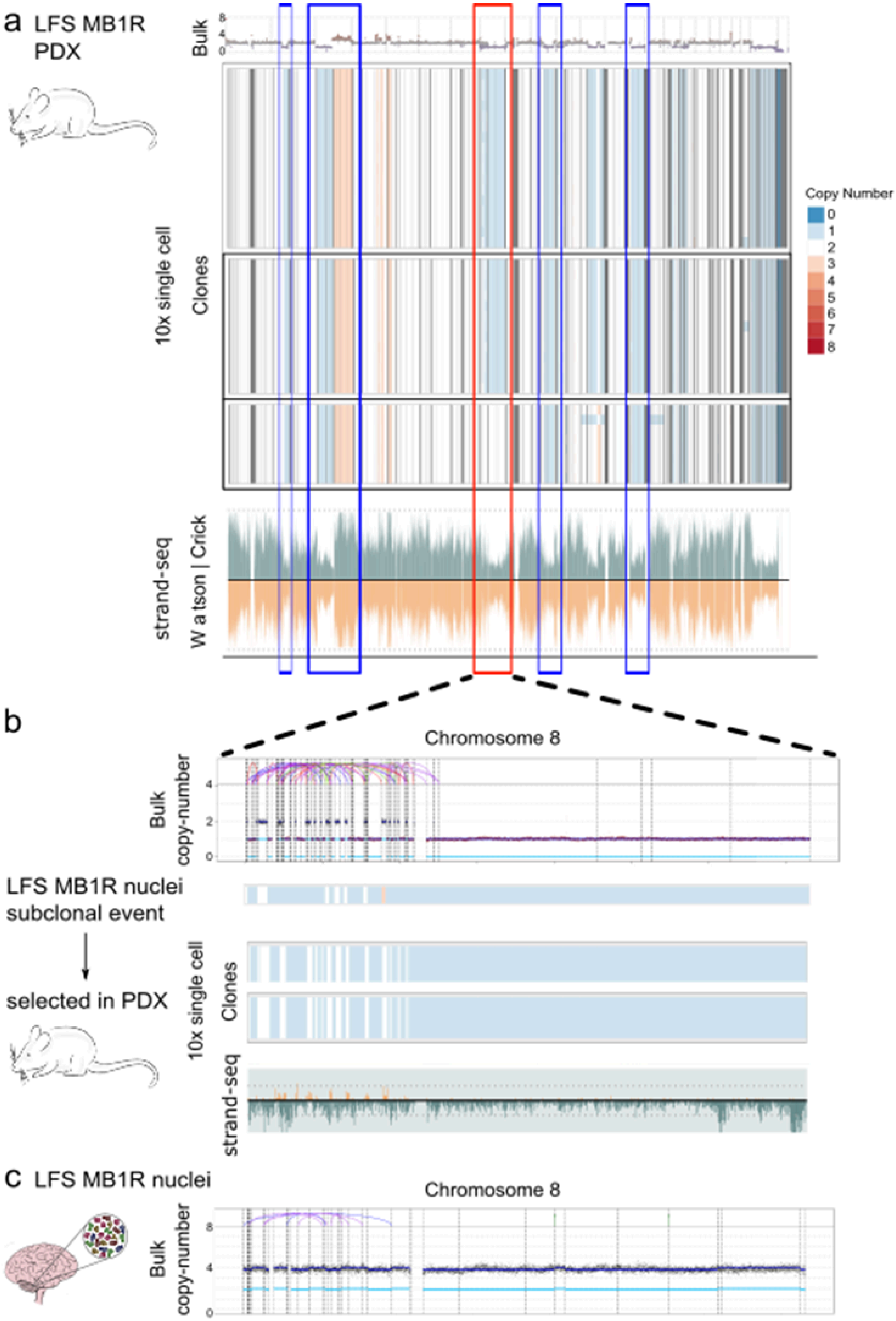

Supplementary Figure 9. Consistency of patterns of oscillating CNV changes, as core hallmark of CT, across single-cell DNA-seq technologies in LFS_MB1R PDX, and evidence for precursor cells in the corresponding primary tumour.

a, Subclonal chromothriptic events in LFS_MB1R recapitulated in strand-seq and bulk WGS. From top to bottom: Copy number from bulk sequencing of the PDX sample; matched single Cell CNV profiles from 10x scDNA sequencing, as inferred by scAbsolute; pseudobulk read counts from strand-seq. Representative regions are highlighted by boxes to highlight concordant copy number changes between the sequencing technologies. Chromosome 8 is highlighted in red as a region with a chromothriptic event detected in the majority of the cells in the PDX sample, but absent in the minor clone (bottom of heatmap).

b, Top to Bottom: SVs and CNV profiles from Chromosome 8 from the LFS_MB1R Nuclei sample; a single cell with a similar CNV profile from the primary tumour (copy state inferred at 20kb resolution using HMMCopy); clonal CNV profiles from two of the clones in LFS_MB1R PDX sample with similar CNV profiles matching the bulk sequencing; and strand-seq read counts of a single cell showing the same breakpoints throughout the chromothriptic region identified in the bulk WGS and 10x scDNA-seq.

c, Bulk CNV profile of chromosome 8 in the LFS_MB1R Nuclei sample, with subclonal oscillating copy number changes suggestive of CT in the same region as the subclonal CT events identified in LFS_MB1R Nuclei. This indicates the presence of a small subpopulation of cells already harbouring chromothripsis on chromosome 8 in the patient tumour, which is selected to become the major clone in the PDX. Source data are available with this manuscript.

Supplementary Figure 10

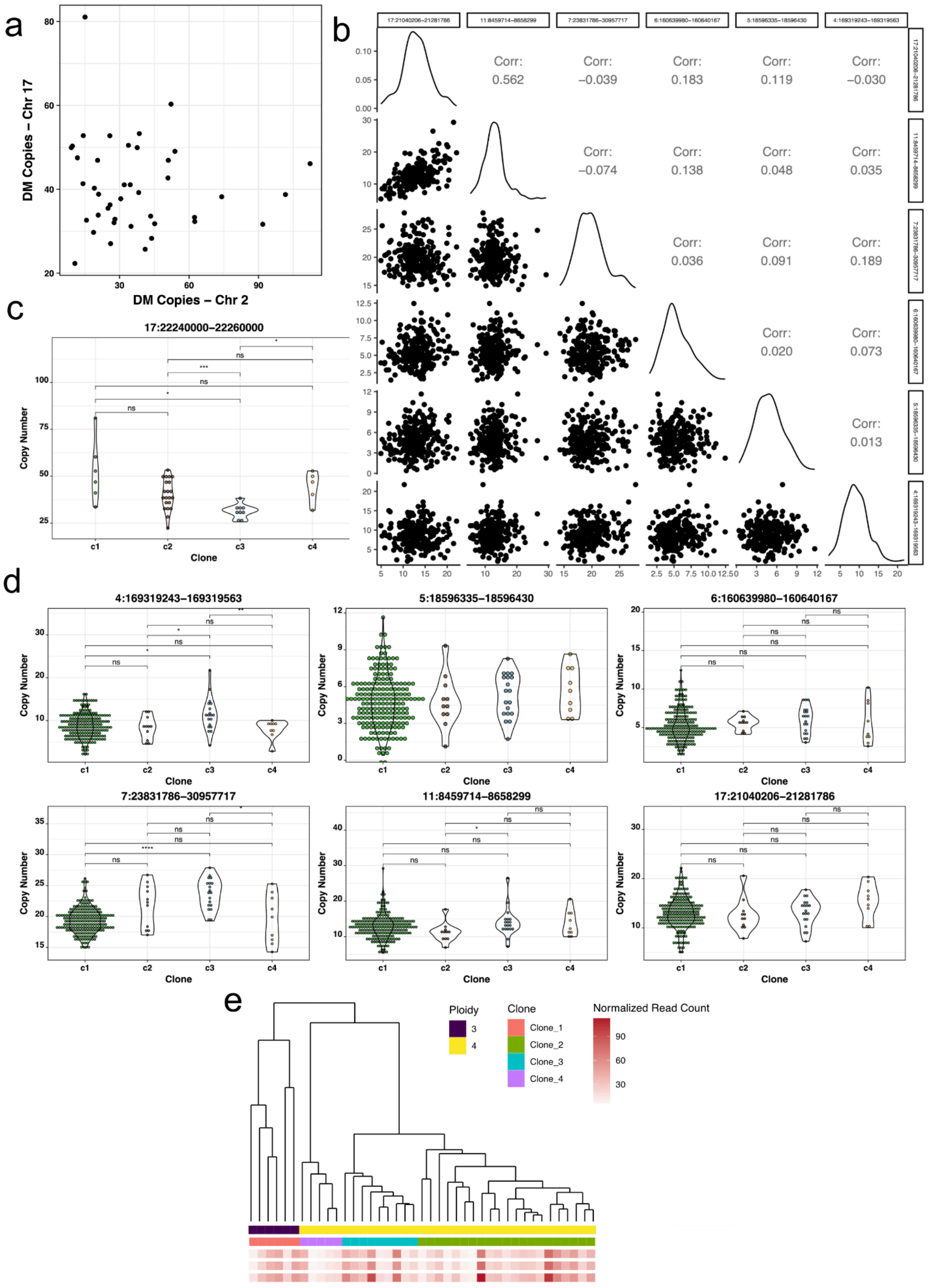

Supplementary Figure 10. Intra-tumor heterogeneity of druggable targets and extrachromosomal circular DNA structures. a, Scatter plot of the normalised read counts for the ecDNA regions on chromosomes 2 and 17 in the MB243 sample. **b,** Scatterplots of normalised read counts (max across bins) for one ecDNA region from each of chromosomes 4, 5, 6, 7, 11, and 17 as identified by AmpliconArchitect per cell across clones in LFS MBP Nuclei. In the case of chromosomes 7, 11, and 17, multiple candidate ecDNA regions were identified, only the region with the highest 97.5% normalised read count across cells is shown for brevity in the lower-triangle**,** with correlations for each pair of ecDNA regions annotated in the upper-triangle, and densities of normalised read counts on the diagonal. **c,** Normalised read counts (max across bins) for the ecDNA region on chromosome 17 in MB243 as identified by AmpliconArchitect per cell across clones, with pairwise differences in mean tested using a two-sided t test, Bonferroni corrected significance annotated. **d,** Normalised read counts (max across bins) for the ecDNA regions in **b** per cell across clones, with pairwise differences in mean tested using a two-sided t test, Bonferroni corrected significance annotated. **e,** Dendrogram after performing ploidy-segregated hierarchical clustering on cells from the MB243-Nuclei sample (Methods). Every leaf in the dendrogram represents a cell, with the cell ploidy, assigned clone, and maximum, mean, and median values across all bins falling within the ecDNA region on chromosome 2 annotated for each cell. Source data are available with this manuscript.

Supplementary Figure 11

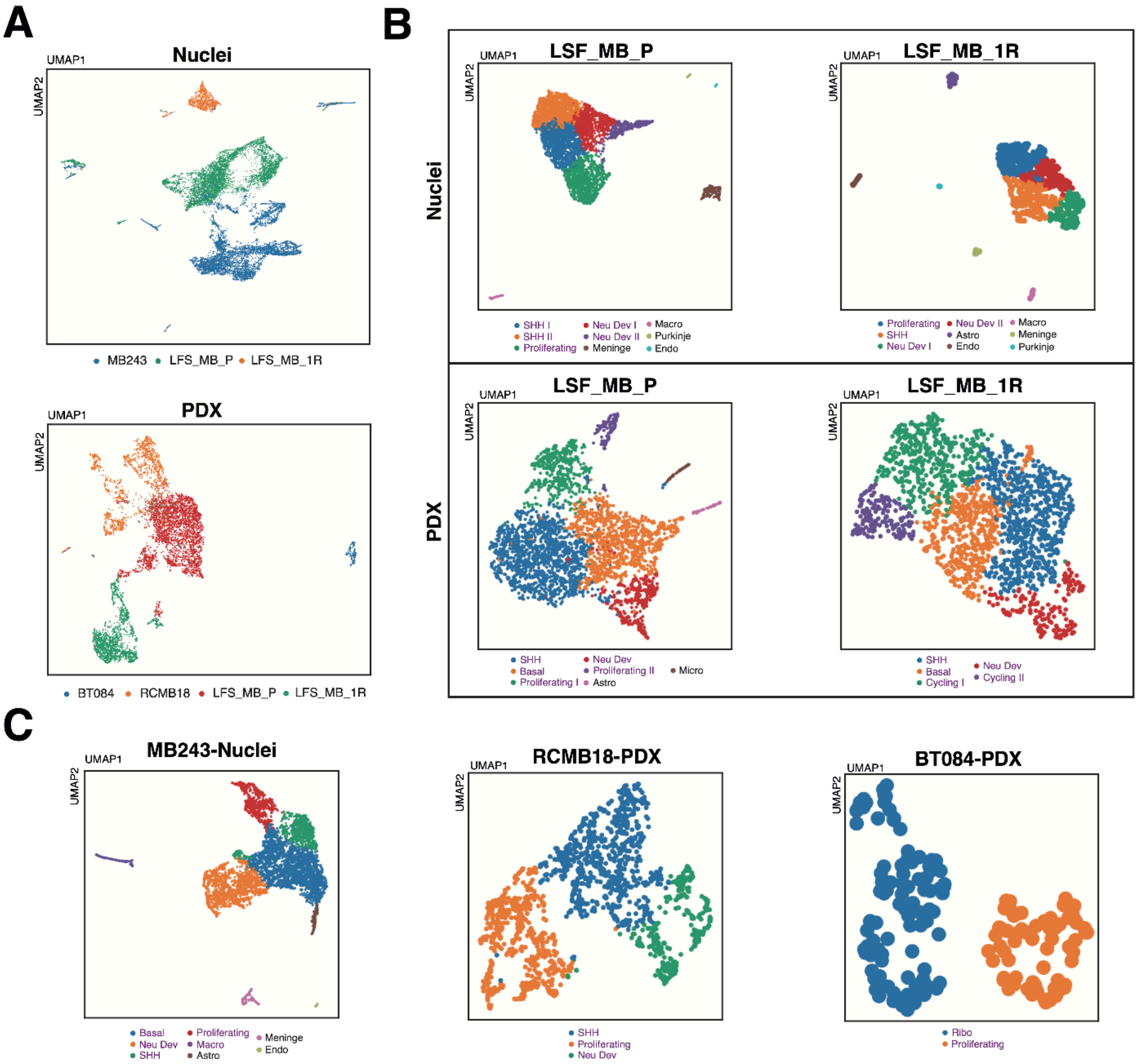

Supplementary Figure 11. UMAP plots of all analysed samples depicting cell type identities. a, UMAP co-embedding from single-nuclei (n=3 samples) and single-cell (n=4 samples) RNA-sequencing highlighting cells derived from each individual sample. **b,** UMAP embeddings for each individual sample acquired from the main patient of the paper (LFS-MB, including LFS-MBP Nuclei, 6,669 cells; PDX, 3,629 cells; and LFS-MB1R Nuclei, 1,322 cells; PDX, 1,817 cells). All cells are coloured according to their cell type assignment. **c,** UMAP plots for each individual sample acquired from additional patients (MB243-Nuclei, 7,302 cells; RCMB18-PDX, 1,613 cells; BT084-PDX, 182 cells). Clusters were assigned to cell types based on markers from the literature (see Methods). Source data are available with this manuscript.

Supplementary Figure 12

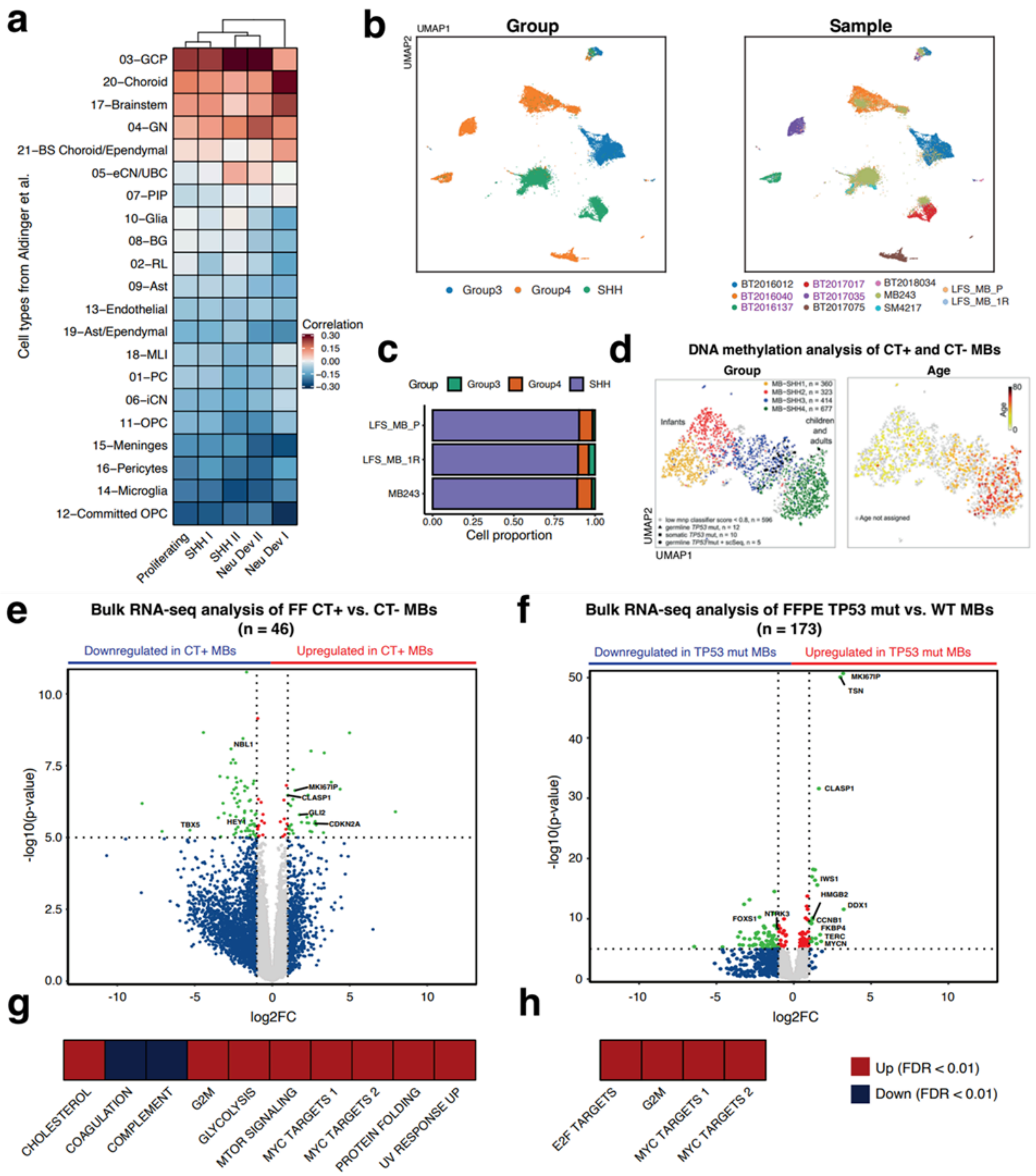

Supplementary Figure 12. Transcriptional signatures of medulloblastomas with chromothripsis. a, Heatmap highlighting the Pearsons' correlation coefficient for each malignant cell type detected in single-nuclei RNA-sequencing from primary tumours compared to the transcriptome of cell types determined by Aldinger et al.[2] **b**, UMAP embedding showing a projection analysis of primary tumour samples used in this study onto the dataset generated by Vladoiu et al[3] (samples BT2016012 to BT2017075, n=8). **c,** Barplot depicting the proportion of cells from (**b**) assigned to a specific MB group from Vladoiu et al.[3] **d,** UMAP embedding results from the analysis of DNA methylation patterns (n=2370) suggesting that CT MBs developing in LFS patients represent a group between infant and children SHH medulloblastomas, together with SHH MBs showing somatic *TP53* mutations. The plot on the left-hand side is coloured according to SHH groups, while the right-hand side shows the age distribution across samples. **e**, Volcano plot, displaying log2 fold change (x-axis) versus negative log p-values (y-axis) of genes identified from a differential expression analysis between fresh frozen (FF) chromothriptic (CT) and non-CT tumours (n = 46; multivariate analysis adjusting for tumour cell content and SHH group; Methods). Vertical and horizontal lines correspond to absolute log2 fold change values of 1 and p < 1E-5 respectively. **f**, Equivalent plot to (**e**) using formalin-fixed paraffin embedded (FFPE) medulloblastomas with *TP53* mut and WT status (n = 173). **g-h**, Gene Set Enrichment Analysis (GSEA) on FF (**g**) and FFPE (**h**) significantly differentially expressed genes shown in (**e**) and (**f**) (FDR < 0.05, Kolmogorov–Smirnov statistic, Benjamini Hochberg adjusted). Source data are available with this manuscript.

Supplementary Figure 13

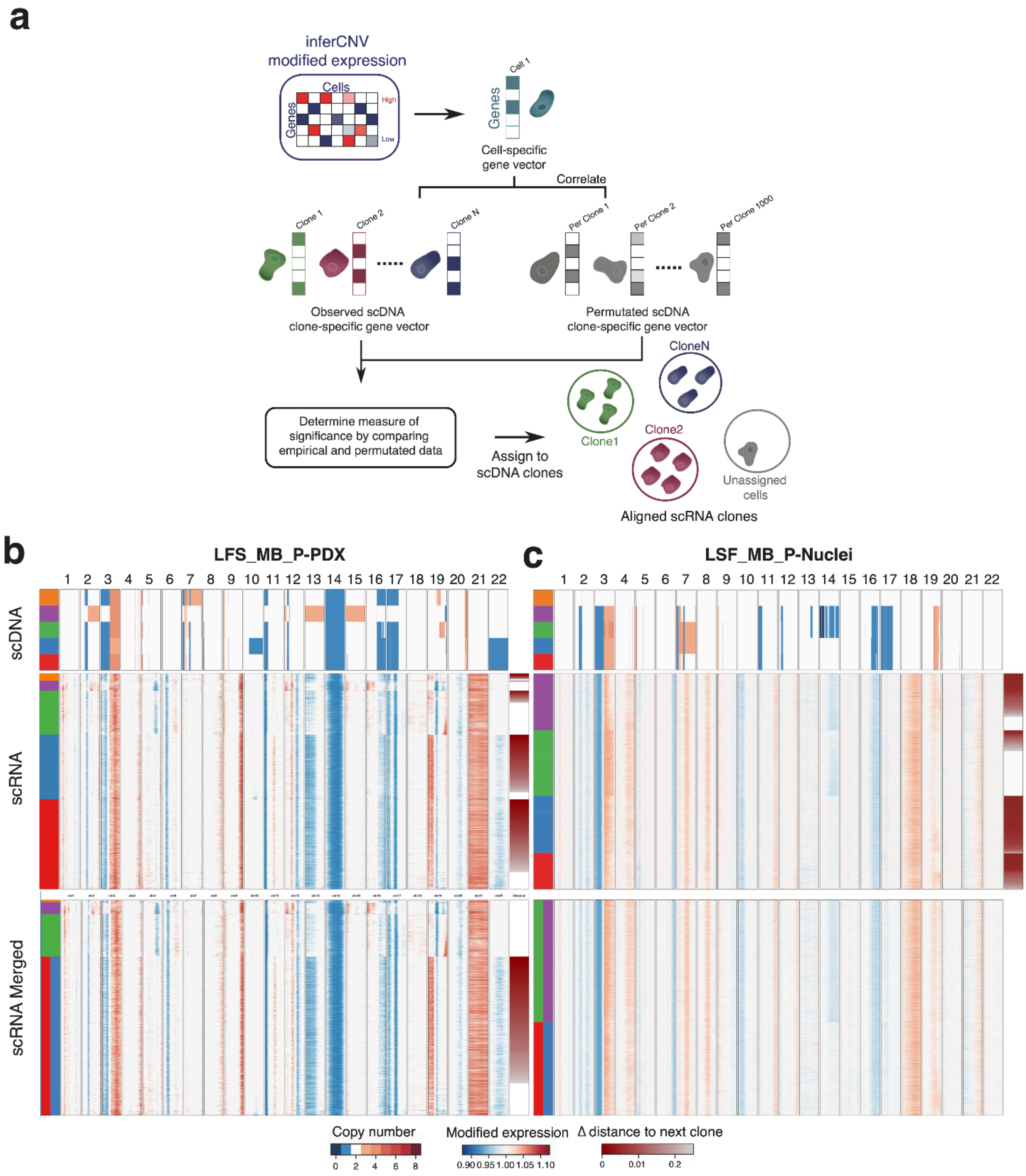

Supplementary Figure 13. Integrated copy number heatmaps from genomic and transcriptomic data. a, Schematic of the computational strategy for aligning scRNA-seq profiles to CNV profiles of clones derived from scDNA-seq. **b-c,** Results from scDNA- and scRNA-seq copy number analysis across all samples. The figure shows the scDNA copy number heatmap at the top, the scRNA-seq copy number for the original mapping, inferred from inferCNV, in the middle, and the scRNA-seq copy number with merged clones presented at the bottom for each sample. No merged-clone heatmap is shown for RCMB18 as only a single clone was detected in the scDNA. Red represents high copy values, white is showing a diploid copy number status and blue depicts low copy number. The scale for scDNA-seq is cut off at copy number values higher than eight (interval = [0,8]). The modified expression scales for inferCNV heatmaps are limited to [0.95, 1.05] with red representing high modified gene expression, white showing a diploid modified gene expression value of 1 and blue indicating low modified gene expression. The colour bar at the left-hand side relates to the clones detected in scDNA for the top heatmap, and the mapped clones for the bottom two heatmaps. Cells are clustered hierarchically within each clone boundary. Of note, instead of showing copy number values per segment, as shown in Figure 1, we here present as transformed to gene-level. Source data are available with this manuscript.

Supplementary Figure 14

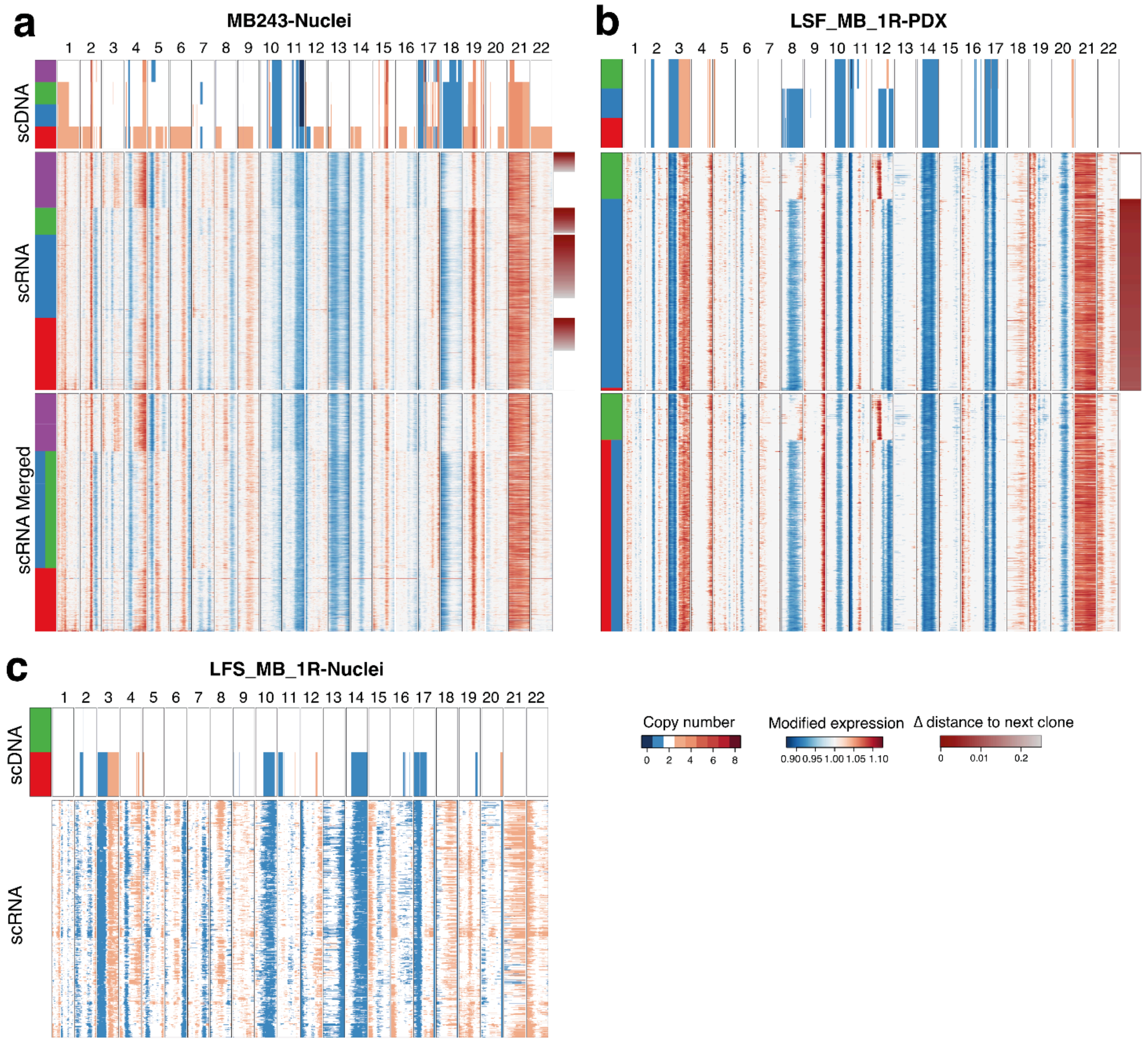

Supplementary Figure 14. Integrated copy number heatmaps from genomic and transcriptomic data. a-c, Results from scDNA- and scRNA-seq copy number analysis across all samples. The figure shows the scDNA copy number heatmap at the top, the scRNA-seq copy number for the original mapping, inferred from inferCNV, in the middle, and the scRNA-seq copy number with merged clones presented at the bottom for each sample. No merged-clone heatmap is shown for RCMB18 as only a single clone was detected in the scDNA. Red represents high copy values, white is showing a diploid copy number status and blue depicts low copy number. The scale for scDNA-seq is cut off at copy number values higher than eight (interval = [0,8]). The modified expression scales for inferCNV heatmaps are limited to [0.95, 1.05] with red representing high modified gene expression, white showing a diploid modified gene expression value of 1 and blue indicating low modified gene expression. The colour bar at the left-hand side relates to the clones detected in scDNA for the top heatmap, and the mapped clones for the bottom two heatmaps. Cells are clustered hierarchically within each clone boundary. Of note, instead of showing copy number values per segment, as shown in Figure 1, we here present as transformed to gene-level. Source data are available with this manuscript.

Supplementary Figure 15

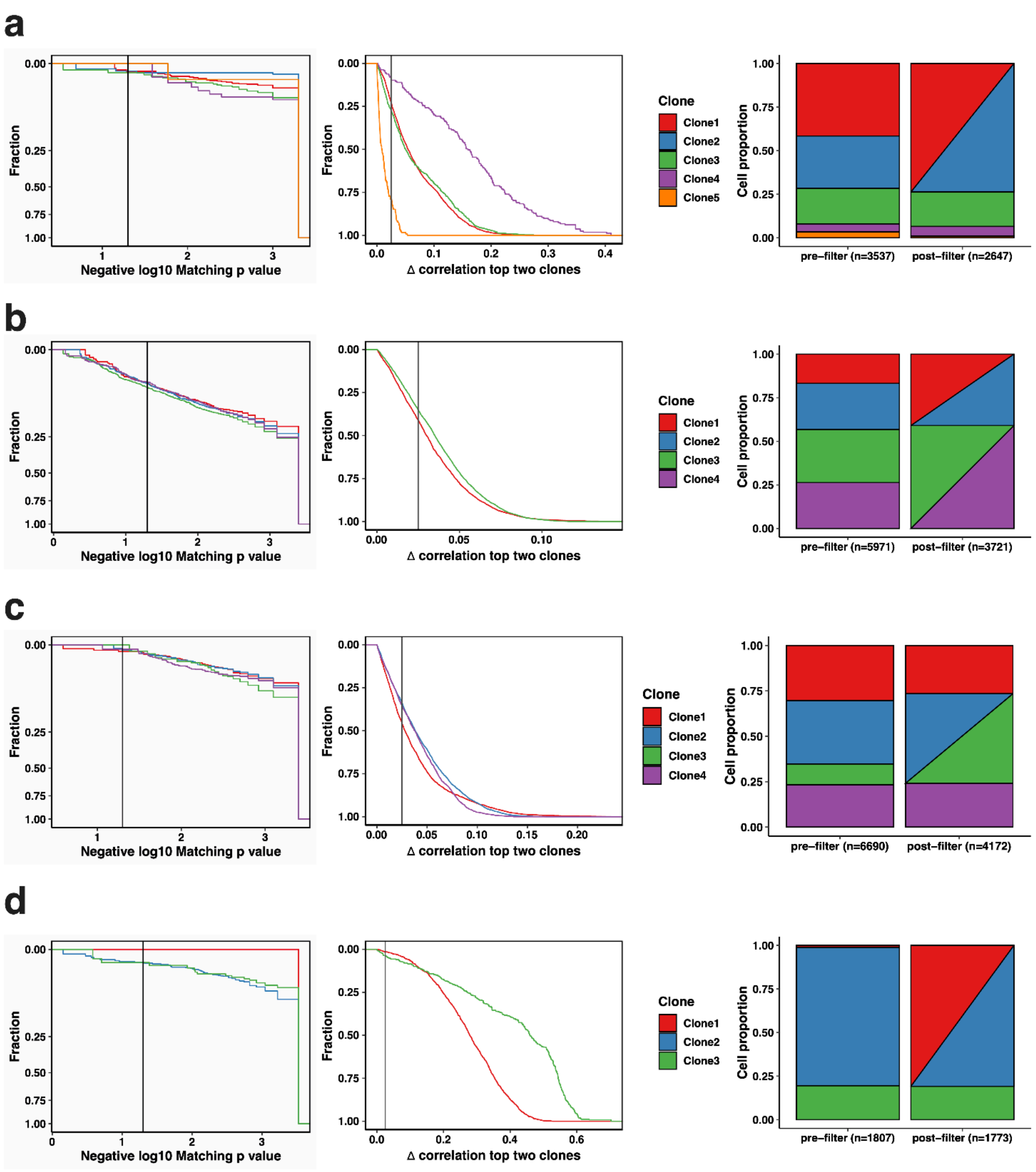

Supplementary Figure 15. Summary of scRNA to scDNA-seq clone alignment. Left to right: Cumulative distribution displaying the fraction of non-aligned cells as a function of the significance threshold (-log10 adjusted p value) for correlation between individual cell CNV profiles, as inferred by inferCNV, and the CNV profile of the best matching DNA clone (calculated through comparison to the background distribution for random clonal CNV profiles). The vertical lines line denotes the threshold significance used in our study; similar cumulative distribution plot for percent cells removed for each difference in correlation between the best and second best clone (after clone merging), with the black vertical line showing the threshold used; and the proportion of all cells in the scRNA-seq assigned to each DNA clone before and after cell filtering and scRNA clone merging steps; for samples **a:** LFS MBP PDX**, b:** LFS MBP Nuclei**, c:** MB243**, d:** LFS MB1R PDX. Source data are available with this manuscript.

Supplementary Figure 16

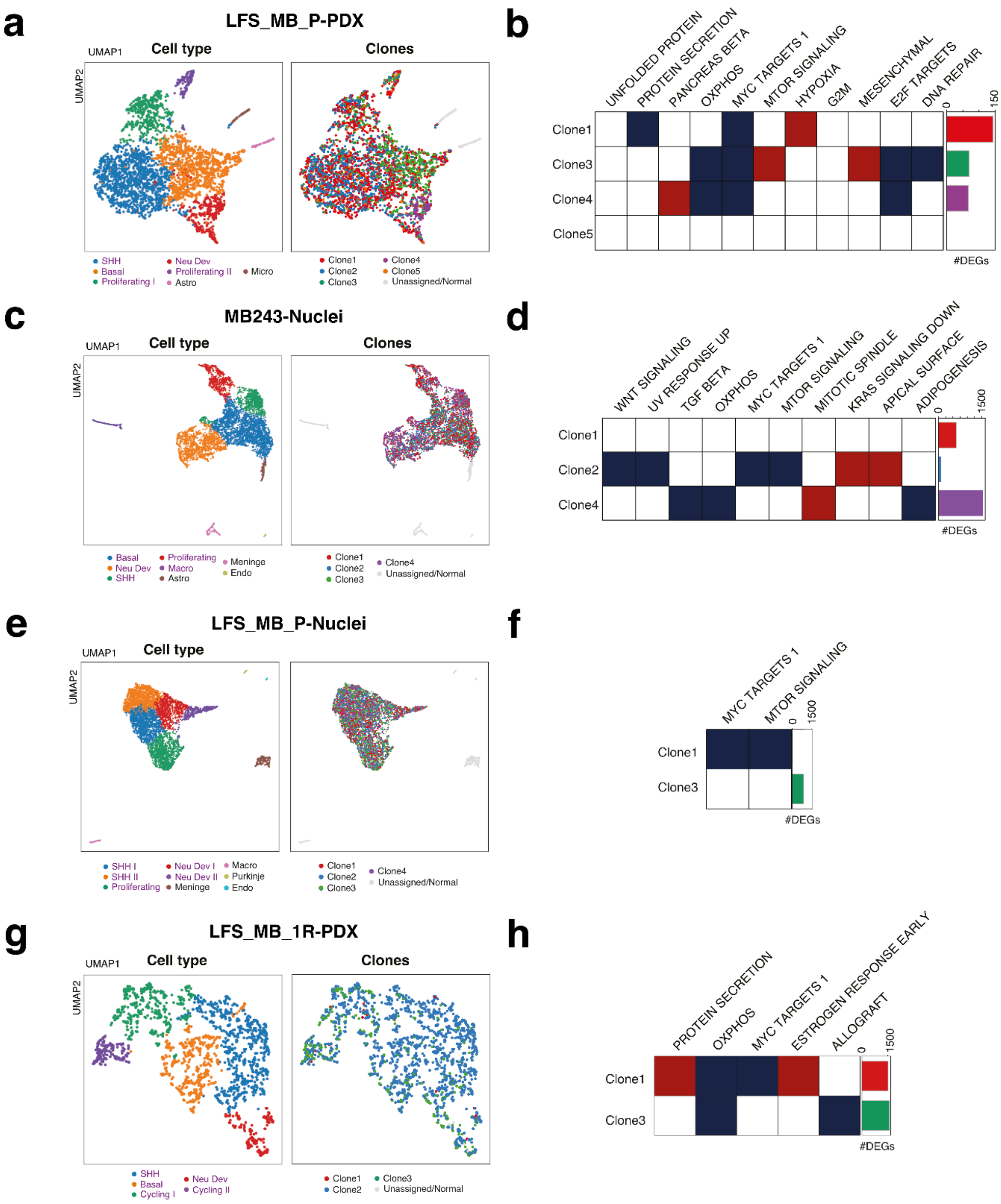

Supplementary Figure 16. Comparisons across clones and downstream analyses of the copy number integration of scRNA- and scDNA-seq data. Results from the copy number integration and downstream transcriptomic analysis for all samples with matched scRNA- and scDNA-seq data. **a,** UMAP embedding depicting cells from scRNA-seq after QC for LFS-MBP PDX. The plot shows the individual cells coloured according to the cell type identity (left) as well as clones (right) from the integrated analysis. Phenotypically normal cells as well as cells which exceeded a FDR > 0.05 were excluded and are marked as unassigned or normal. **b,** Heatmap for GSEA for each individual clone detected in scRNA-seq. Additionally, the number of significantly differentially expressed genes comparing the cells of a specific clone with all other cells is shown as a bar plot on the top. Genes are considered significantly differentially expressed if they have a FDR < 0.05 (Wilcoxon rank sum test, Benjamini Hochberg adjusted, one clone versus all). Each bar is coloured based on the clone colour shown in **a**. For the pathways, significance is shown as FDR < 0.05 (Kolmogorov–Smirnov statistic, Benjamini Hochberg adjusted). Depending on the type of differential expression (up- or down regulation), the pathways are shown in shades of red or blue. As for the druggable targets in Figure 3, clones with ambiguous cell assignment have been merged. **c-d**, Equivalent plots for MB243 Nuclei. **e-f**, Equivalent plots for LFS-MBP Nuclei. **g-h**, Equivalent plots for LFS-MB1R PDX. Source data are available with this manuscript.

Supplementary Figure 17

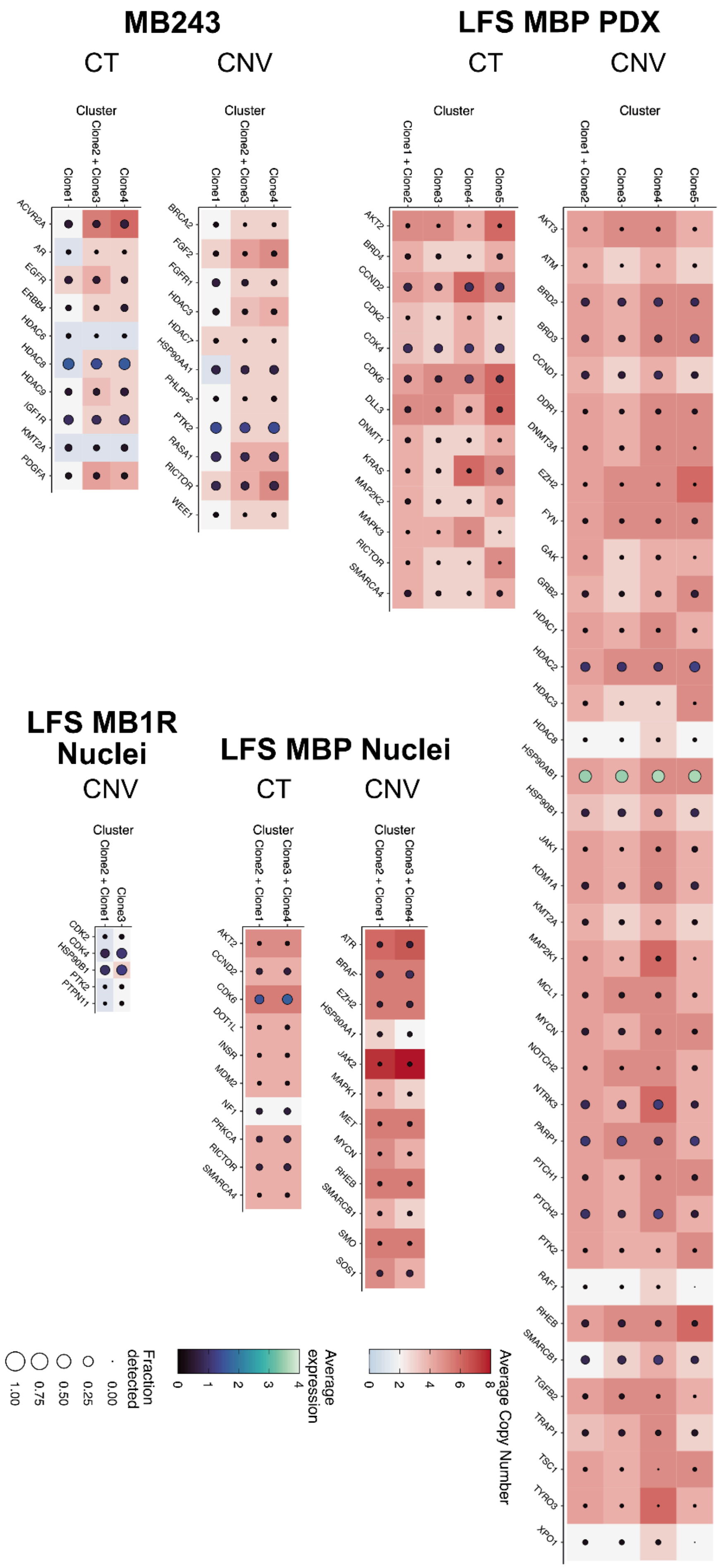

Supplementary Figure 17. Genomic and transcriptomic heterogeneity across druggable targets in single-cell sequencing. Dotplots showing the copy number status from scDNA-seq (background colour) and expression from scRNAseq (dot colour) of selected druggable targets in all clones. Dot size is proportional to the percentage of cells per clone for which the gene expression is detected. Source data are available with this manuscript.

Supplementary Figure 18

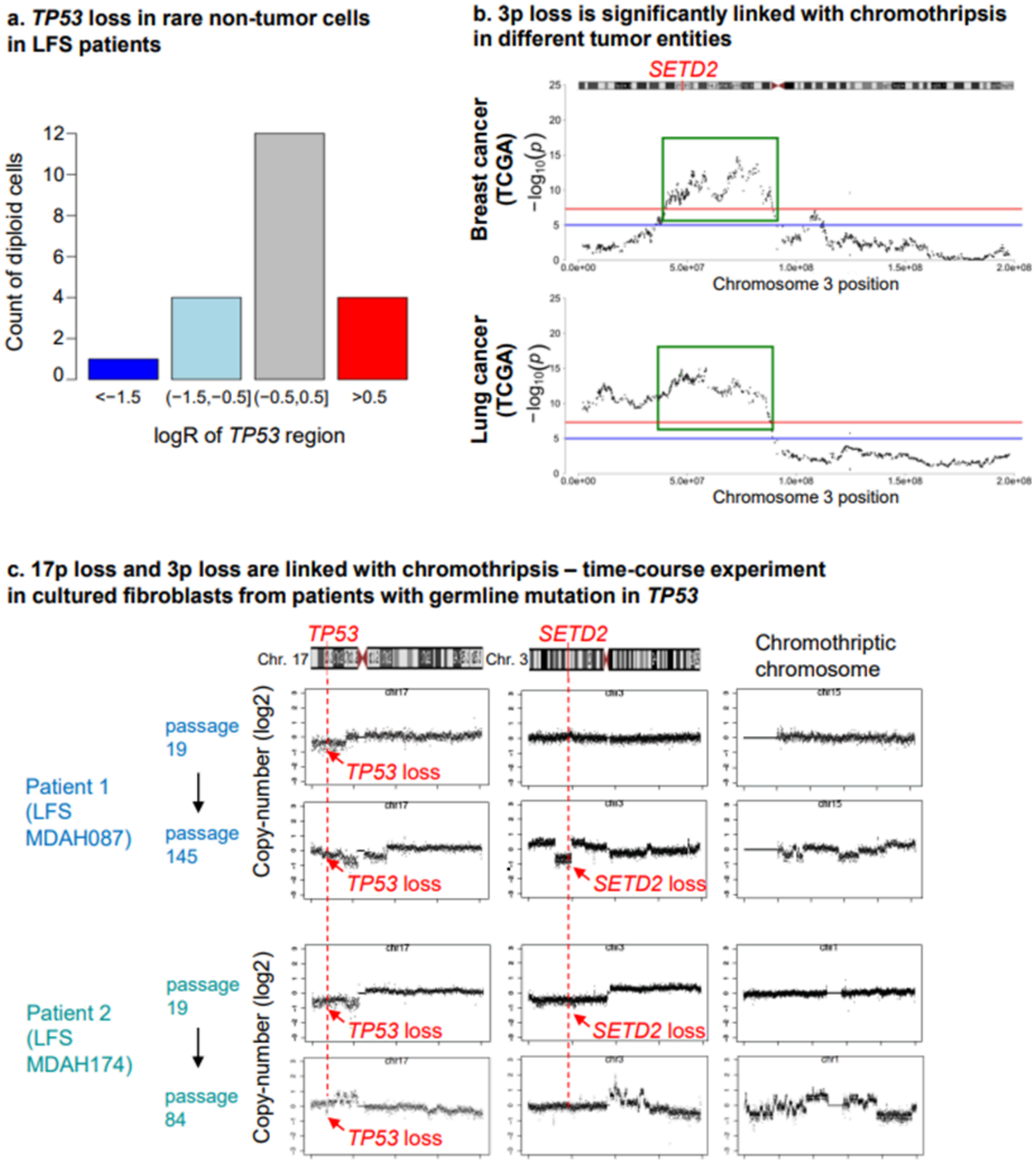

Supplementary Figure 18 Loss of chr3p and chr17p as early events linked to chromothripsis. a, *TP53* loss in rare non-tumor cells from LFS-MBP Nuclei sample. **b**, Chromosome 3p loss is significantly linked with chromothripsis in different tumour entities (re-analysis of TCGA data)[4]. **c**, 17p and 3p loss are linked with chromothripsis - time course experiment in cultured fibroblasts from patients with germline mutation in *TP53*. Source data are available with this manuscript.

Supplementary Figure 19

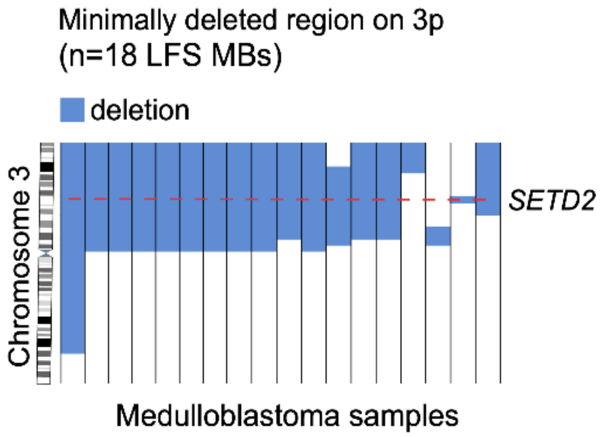

Supplementary Figure 19. *SETD2* is located in the minimally deleted region on chromosome 3p in LFS medulloblastoma. N=18. Blue shades show the size and location of the deleted region. Each vertical bar shows one medulloblastoma with chromothripsis.

Supplementary Figure 20

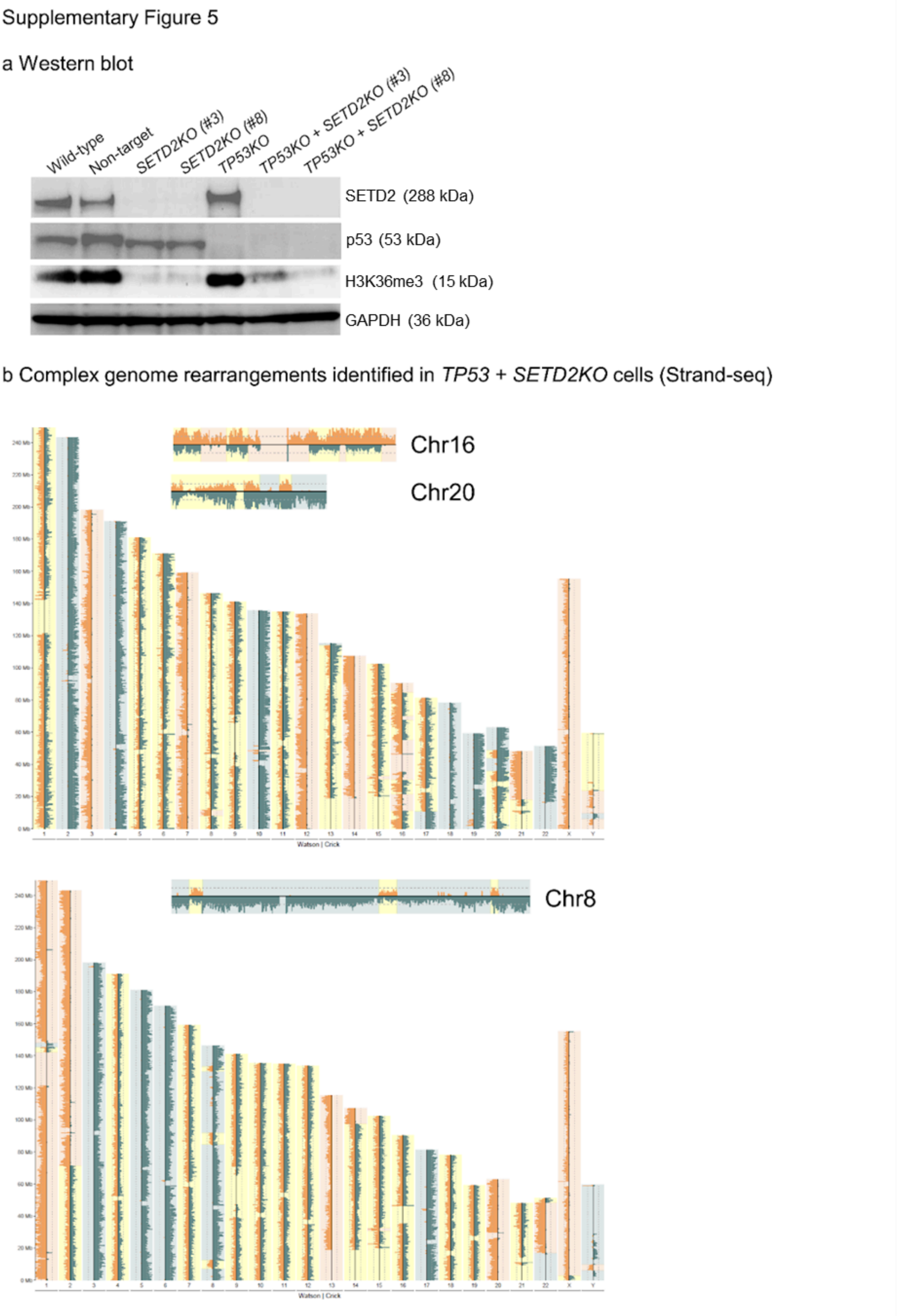

Supplementary Figure 20. **Inactivation of *SETD2* and *TP53* in neural stem cells leads to genome instability. a**, Western blot analysis confirms efficient inactivation of *SETD2* and *TP53* in neural stem cells by CRISPR/Cas9. Western blotting was repeated three times with similar results. Two independent guide RNAs were used for *SETD2* (guides #3 and #8). **b**, Strand-seq analysis of neural stem cells after inactivation of *SETD2* and *TP53* shows

structural variants, including complex rearrangements. Source data are available with this manuscript.

Original uncropped scans of Western blot shown in Supplementary Figure 20a

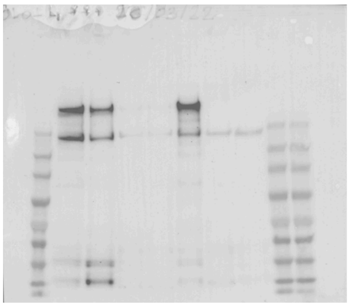
SETD2 (288 kDa)

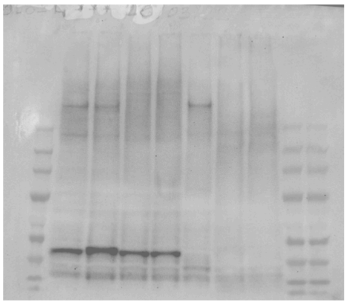
P53 (53 kDa)

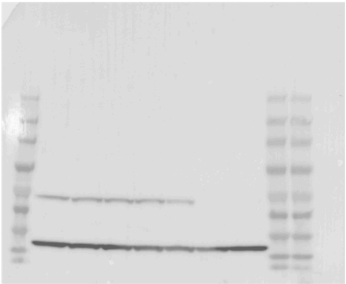
GAPDH (36 kDa)

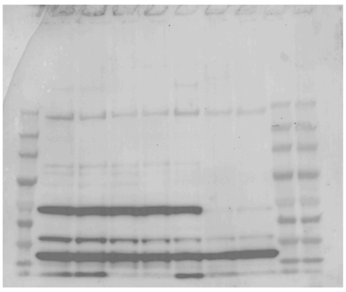
H3K36me3 (15 kDa)

Supplementary References

1. Umbreit, N. T. *et al.* Mechanisms generating cancer genome complexity from a single cell division error. *Science* **368**, eaba0712 (2020).
2. Aldinger, K. A. *et al.* Spatial and cell type transcriptional landscape of human cerebellar development. *Nat. Neurosci.* **24**, 1163–1175 (2021).
3. Vladoiu, M. C. *et al.* Childhood cerebellar tumours mirror conserved fetal transcriptional programs. *Nature* **572**, 67–73 (2019).
4. Hoadley, K. A. *et al.* Cell-of-Origin Patterns Dominate the Molecular Classification of 10,000 Tumors from 33 Types of Cancer. *Cell* **173**, 291-304.e6 (2018).
