# Supplementary material for: Multi-omic and single-cell profiling of chromothriptic medulloblastoma reveals genomic and transcriptomic consequences of genome instability
Source: Nat Commun. 2024 Nov 23;15:10183. doi: 10.1038/s41467-024-54547-w (PMC11585558; doi:10.1038/s41467-024-54547-w)
Supplement: Supplementary file 2 — Description of Additional Supplementary Information [file 41467_2024_54547_MOESM2_ESM.docx]

**Description of Additional Supplementary Files**

File Name: Supplementary Data 1

Description: Overview of quality control and filtering criteria in scDNA-seq.

File Name: Supplementary Data 2

Description: Chromothripsis scoring comparison between bulk WGS and single-cell DNA-seq. scDNA-seq chromothripsis status per chromosome called as described in Methods; for bulk WGS, scoring using Shatterseek as well as by visual inspection is included.

File Name: Supplementary Data 3

Description: Overview of the number of cells assigned to each scDNA-seq clone in the study.

File Name: Supplementary Data 4

Description: Overview of quality control and filtering criteria in scRNA-seq.

File Name: Supplementary Data 5

Description: Marker gene expression for clusters in scRNA-seq used for annotating cell types. Significance reported from two sided Mann-Whitney-U test, Benjamini-Hochberg adjusted.

File Name: Supplementary Data 6

Description: Results of the bulk RNA-seq differential gene expression analysis using DESeq2. Tab 1 is the comparison of FF CT+ to CT- samples. Tab 2 is the comparison of FFPE TP53mut vs TP53wt samples. Significance was assessed using a 2-sided Wald test and FDR is reported.

File Name: Supplementary Data 7

Description: Overview of the results of mapping scRNA-seq to scDNA-seq clones.

File Name: Supplementary Data 8

Description: Differential expression analysis between clones. Significance reported from two sided Mann-Whitney-U test, Benjamini-Hochberg adjusted.
