# Supplementary material for: Multi-omic and single-cell profiling of chromothriptic medulloblastoma reveals genomic and transcriptomic consequences of genome instability
Source: Nat Commun. 2024 Nov 23;15:10183. doi: 10.1038/s41467-024-54547-w (PMC11585558; doi:10.1038/s41467-024-54547-w)
Supplement: Supplementary file 11 — Reporting Summary [file 41467_2024_54547_MOESM11_ESM.pdf]

nature portfolio

Corresponding author(s): Oliver Stegle and Aurélie Ernst

Last updated by author(s): Oct 10, 2024

Reporting Summary

Nature Portfolio wishes to improve the reproducibility of the work that we publish. This form provides structure for consistency and transparency in reporting. For further information on Nature Portfolio policies, see our Editorial Policies and the Editorial Policy Checklist.

Statistics

For all statistical analyses, confirm that the following items are present in the figure legend, table legend, main text, or Methods section.

n/a	Confirmed	
☐	☒	The exact sample size (*n*) for each experimental group/condition, given as a discrete number and unit of measurement
☐	☒	A statement on whether measurements were taken from distinct samples or whether the same sample was measured repeatedly
☐	☒	The statistical test(s) used AND whether they are one- or two-sided<br>*Only common tests should be described solely by name; describe more complex techniques in the Methods section.*
☐	☒	A description of all covariates tested
☐	☒	A description of any assumptions or corrections, such as tests of normality and adjustment for multiple comparisons
☐	☒	A full description of the statistical parameters including central tendency (e.g. means) or other basic estimates (e.g. regression coefficient) AND variation (e.g. standard deviation) or associated estimates of uncertainty (e.g. confidence intervals)
☐	☒	For null hypothesis testing, the test statistic (e.g. *F*, *t*, *r*) with confidence intervals, effect sizes, degrees of freedom and *P* value noted<br>*Give P values as exact values whenever suitable.*
☐	☒	For Bayesian analysis, information on the choice of priors and Markov chain Monte Carlo settings
☐	☒	For hierarchical and complex designs, identification of the appropriate level for tests and full reporting of outcomes
☐	☒	Estimates of effect sizes (e.g. Cohen's *d*, Pearson's *r*), indicating how they were calculated

Our web collection on statistics for biologists contains articles on many of the points above.

Software and code

Policy information about availability of computer code

Data collection	*Provide a description of all commercial, open source and custom code used to collect the data in this study, specifying the version used OR state that no software was used.*
Data analysis	The main published and widely available software packages used in the manuscript are:<br>-Scanpy<br>-HMMCopy<br>-inferCNV<br>-scAbsolute<br>-10x Cell Ranger<br>- Shatterseek<br><br>Custom codes are provided in the github repository cited in the manuscript.

For manuscripts utilizing custom algorithms or software that are central to the research but not yet described in published literature, software must be made available to editors and reviewers. We strongly encourage code deposition in a community repository (e.g. GitHub). See the Nature Portfolio guidelines for submitting code & software for further information.

Data

Policy information about availability of data

All manuscripts must include a data availability statement. This statement should provide the following information, where applicable:

- Accession codes, unique identifiers, or web links for publicly available datasets
- A description of any restrictions on data availability
- For clinical datasets or third party data, please ensure that the statement adheres to our policy

Raw sequencing data from single cell WGS, single cell RNAseq, and Strand-Seq experiments generated in this study are deposited with the European Genome-phenome Archive (EGA), which is hosted by the EBI and the CRG, under accession number EGAS00001005410 [https://ega-archive.org/studies/EGAS00001005410]. The data are available under restricted access due to the European General Data Protection Regulation (GDPR) and the German General Data Protection Regulation (GDPR) and to respect the patient consent forms. Data access can be requested through the EGA subject to Data Access Committee review. It can be granted in principle for research use after a Data Transfer Agreement is legally settled between the requesting institute and the providing institute. Once the data access has been granted, the access is usually available for 5 years, unless otherwise restricted by individual patient consent forms. Data access requests will be reviewed and Data Transfer Agreements will be settled as quickly as possible.

Processed sequencing data for WGS/WES, scDNA, scRNA and StrandSeq assays are available on Zenodo under the doi: 10.5281/zenodo.13348419 [http://doi.org/10.5281/zenodo.13348419]. The samples profiled in this study are embedded in the larger ICGC PedBrain project, and raw sequencing data for all analyses of bulk short read WGS and RNA sequencing for the PedBrain samples are available after through the EGA under accession number EGAS00001001953 [https://www.ebi.ac.uk/ega/studies/EGAS00001001953]40. Raw RNAseq data used for differential expression analysis in this study from Waszal et al. is available through the EGA under accession number EGAS00001004126 [https://www.ebi.ac.uk/ega/studies/EGAS00001004126]41 and from Kool et al. from the EGA under accession number EGAS00001000607 [https://www.ebi.ac.uk/ega/studies/EGAS00001000607]42. scRNAseq data from Reimondy et al. use in this manuscript are available from GEO under SuperSeries accession number GSE156053 [https://www.ncbi.nlm.nih.gov/geo/query/acc.cgi?acc=GSE156053] 23. Raw data for scRNAseq from Vladoiu et al. used in this manuscript are available from the EGA under accession code EGAS00001003170 [https://www.ebi.ac.uk/ega/studies/EGAS00001003170] 37. Raw snRNAseq data from Aldinger et al. used in this manuscript are available from dbGaP under accession code phs001908.v2.p1 [https://www.ncbi.nlm.nih.gov/projects/gap/cgi-bin/study.cgi?study_id=phs001908.v2.p1]39. Raw single cell WGS data from Umbreit et al. used in this manuscript are available from the Sequence Read Archive under project code SRP24383216 [https://trace.ncbi.nlm.nih.gov/Traces/?view=study&acc=SRP243832].
The methylation array data from Capper et al. used in this manuscript are available through GEO under accession number GSE10938165 [https://www.ncbi.nlm.nih.gov/geo/query/acc.cgi?acc=GSE109381].

Source data for all figures are accessible through Zenodo under the doi: 10.5281/zenodo.13918598 [http://doi.org/10.5281/zenodo.13918598]. The remaining data are available within the Article, Supplementary Information or in the Source Data files.

Research involving human participants, their data, or biological material

Policy information about studies with human participants or human data. See also policy information about sex, gender (identity/presentation), and sexual orientation and race, ethnicity and racism.

Reporting on sex and gender	Sex of human patients is reported in the manuscript. Gender is not considered in this study.
Reporting on race, ethnicity, or other socially relevant groupings	Race, ethnicity or socially relevant groupings are not reported in the manuscript.
Population characteristics	Cancer patients
Recruitment	Recruitment after informed consent.
Ethics oversight	Clinical samples and data were collected, after receiving written informed consent in accordance with the Declaration of Helsinki and approval from the Heidelberg University.

Note that full information on the approval of the study protocol must also be provided in the manuscript.

Field-specific reporting

Please select the one below that is the best fit for your research. If you are not sure, read the appropriate sections before making your selection.

☒ Life sciences ☐ Behavioural & social sciences ☐ Ecological, evolutionary & environmental sciences

For a reference copy of the document with all sections, see nature.com/documents/nr-reporting-summary-flat.pdf

Life sciences study design

All studies must disclose on these points even when the disclosure is negative.

Sample size	Sample sizes were largely determined by the rarity of the condition studied.
Data exclusions	Sample BT084 was excluded for the scDNAseq analysis due to insufficient quality.

Replication	NA
Randomization	NA
Blinding	NA

Reporting for specific materials, systems and methods

We require information from authors about some types of materials, experimental systems and methods used in many studies. Here, indicate whether each material, system or method listed is relevant to your study. If you are not sure if a list item applies to your research, read the appropriate section before selecting a response.

Materials & experimental systems

n/a	Involved in the study
☐	☒ Antibodies
☐	☒ Eukaryotic cell lines
☒	☐ Palaeontology and archaeology
☐	☒ Animals and other organisms
☒	☐ Clinical data
☒	☐ Dual use research of concern
☒	☐ Plants

Methods

n/a	Involved in the study
☒	☐ ChIP-seq
☒	☐ Flow cytometry
☒	☐ MRI-based neuroimaging

Antibodies

Antibodies used

SETD2 (E4W8Q) Rabbit mAb (Cell signaling, #80290) ;TP53 (DO-1) (Santa Cruz, #sc-126) Mouse mAb , GAPDH (Sigma-Aldrich, CB1001) Mouse mAb (6C5); Anti-gamma H2A.X (phospho S139) (Abcam, # ab11174), Anti-SETD2 antibody (Atlas Antibodies, #HPA042451) Rabbit polyclonal; Acetyl-α-Tubulin (Lys40) (D20G3) XP (Cell Signaling, #5335) Rabbit mAb; Phospho-Histone H3 (Ser10) (6G3) (Cell Signaling, #9706) Mouse mAb, H3K36me3 rabbit pAb (Abcam, ab9050)

Validation

Western Blotting:
SETD2 (E4W8Q) rabbit mAb (Cell Signaling, #80290) (dilution 1:1000)
Lot #1
Species reactivity: Human, Mouse and Monkey
Approved applications: Western Blotting, Chromatin IP and Chromatin IP-seq
This antibody has been validated using SimpleChIP® Enzymatic Chromatin IP Kits.

TP53 (DO-1) mouse mAb (Santa Cruz, #sc-126) (dilution 1:500)
Species reactivity: Human, Mouse and Rat
Applications: FACS, IF, IHC, IP and WB
Reference: Banks, L., et al. 1986. Isolation of human-p53-specific monoclonal antibodies and their use in the studies of human p53 expression. Eur. J. Biochem. 159: 529-534

GAPDH (6C5) mouse mAb (Sigma-Aldrich, CB1001) (dilution 1:2000)
Species reactivity: Human, Mouse, Porcine, Chicken, Canine, Rabbit, Fish, Rat, Frog,
Applications: WB and IF
Reference: Khawaled S, Nigita G, Distefano R, et al. Pleiotropic tumor suppressor functions of WWOX antagonize metastasis. Signal Transduct Target Ther. 2020;5(1):43. Published 2020 Apr 17. doi:10.1038/s41392-020-0136-8

H3K36me3 rabbit pAb (Abcam, ab9050) (dilution 1:400)
Reactivity: Human and Cow
Applications: ChIP, WB, ICC/IF
Reference: Tiedemann, R. L., Hlady, R. A., Hanavan, P. D., Lake, D. F., Tibes, R., Lee, J. H., Choi, M., & Von Hoff, D. D. (2016). Dynamic reprogramming of DNA methylation in SETD2-deregulated renal cell carcinoma. Oncotarget, 7(2), 1927-1946. https://doi.org/10.18632/oncotarget.6481

Immunohistochemistry
Anti-SETD2 antibody (Atlas Antibodies, #HPA042451) Rabbit polyclonal (dilution 1:500)
Verified species reactivity: Human
Applications: IHC, ICC-IF
This antibody has been used for staining of 44 normal human tissue samples as well as human cancer samples covering the 20 most common cancer types and up to 12 patients for each cancer type.

Immunofluorescence
Anti-gamma H2A.X (phospho S139) (Abcam, # ab11174) Rabbit polyclonal (dilution 1:200)
Reactivity: Mouse and Human

Applications: IHC-P, ICC/IF, WB
Reference: Lambo, Sander et al. "The molecular landscape of ETMR at diagnosis and relapse." Nature vol. 576,7786 (2019): 274-280. doi:10.1038/s41586-019-1815-x

Acetyl-α-Tubulin (Lys40) (D20G3) XP (Cell Signaling, #5335) Rabbit mAb (dilution 1:400)
Lot #5
Reactivity: Human, Mouse, Rat, Monkey and Zebrafish
Applications: IF, IP, WB and Flow cytometry
Reference: Li F, Sawada J, Komatsu M. R-Ras-Akt axis induces endothelial lumenogenesis and regulates the patency of regenerating vasculature. Nat Commun. 2017;8(1):1720. Published 2017 Nov 23. doi:10.1038/s41467-017-01865-x

Phospho-Histone H3 (Ser10) (6G3) (Cell Signaling, #9706) Mouse mAb (dilution 1:200)
Lot #10
Reactivity: Human, Mouse and Rat
Applications: IF, WB and Flow cytometry
Reference: Hendzel MJ, Wei Y, Mancini MA, et al. Mitosis-specific phosphorylation of histone H3 initiates primarily within pericentromeric heterochromatin during G2 and spreads in an ordered fashion coincident with mitotic chromosome condensation. Chromosoma. 1997;106(6):348-360. doi:10.1007/s004120050256

Eukaryotic cell lines

Policy information about cell lines and Sex and Gender in Research

Cell line source(s)	Neural stem cells kindly provided by Dr Daniel Haag; the sample was male.
Authentication	These cells are derived from iPSCs, they cannot be authenticated like common cell lines
Mycoplasma contamination	Cells were tested negative for mycoplasma contamination.
Commonly misidentified lines (See ICLAC register)	*Name any commonly misidentified cell lines used in the study and provide a rationale for their use.*

Animals and other research organisms

Policy information about studies involving animals; ARRIVE guidelines recommended for reporting animal research, and Sex and Gender in Research

Laboratory animals	Immunocompromised mice were used for the patient-derived xenografts (6-10-week-old female immune-compromised mice (NSG, NOD.Cg-PrkdcscidIl2rgtm1Wjl)
Wild animals	NA
Reporting on sex	NA
Field-collected samples	NA
Ethics oversight	All animal experiments were performed in accordance with ethical and legal regulations for animal welfare and approved by the governmental council (Regierungspräsidium Karlsruhe, Germany).

Note that full information on the approval of the study protocol must also be provided in the manuscript.

Plants

Seed stocks	*Report on the source of all seed stocks or other plant material used. If applicable, state the seed stock centre and catalogue number. If plant specimens were collected from the field, describe the collection location, date and sampling procedures.*
Novel plant genotypes	*Describe the methods by which all novel plant genotypes were produced. This includes those generated by transgenic approaches, gene editing, chemical/radiation-based mutagenesis and hybridization. For transgenic lines, describe the transformation method, the number of independent lines analyzed and the generation upon which experiments were performed. For gene-edited lines, describe the editor used, the endogenous sequence targeted for editing, the targeting guide RNA sequence (if applicable) and how the editor was applied.*
Authentication	*Describe any authentication procedures for each seed stock used or novel genotype generated. Describe any experiments used to assess the effect of a mutation and, where applicable, how potential secondary effects (e.g. second site T-DNA insertions, mosiacism, off-target gene editing) were examined.*
